# Supplementary material for: GABAA Receptor-Stabilizing Protein Ubqln1 Affects Hyperexcitability and Epileptogenesis after Traumatic Brain Injury and in a Model of In Vitro Epilepsy in Mice
Source: Int J Mol Sci. 2022 Mar 31;23(7):3902. doi: 10.3390/ijms23073902 (PMC8999075; doi:10.3390/ijms23073902)
Supplement: Supplementary file 1 [file ijms-23-03902-s001.zip › Supplementary Materials-doi10.3390ijms23073902.pdf]

Supplementary Materials

GABAA Receptor-Stabilizing Protein Ubqln1 Affects Hyperexcitability and Epileptogenesis after Traumatic Brain Injury and in a Model of In Vitro Epilepsy in Mice

Tabea Kürten [1,†], Natascha Ihbe [1,†],Timo Ueberbach [1], Ute Distler [2], Malte Sielaff [2], Stefan Tenzer [2] and Thomas Mittmann [1,*]

1 Institute of Physiology, University Medical Center of the Johannes Gutenberg University Mainz, Duesbergweg 6, 55128 Mainz, Germany
2 Institute for Immunology, University Medical Center of the Johannes Gutenberg University Mainz, Langenbeckstraße 1, 55131 Mainz, Germany
* Author to whom correspondence should be addressed.
† These authors contributed equally to the work.

UniProt ID	Accession	FDR	p-Value (adjusted) sham vs contralateral GFP+
TBA3_MOUSE	P05214	0	1E-12
NFL_MOUSE	P08551	0	1E-12
ARL11_MOUSE	Q6P3A9	0	1E-12
ACBG1_MOUSE	Q99PU5	0	0,01328243
VPS4B_MOUSE	P46467	0.0008	1E-12
K1C24_MOUSE	A1L317	0	1E-12
H2A2C_MOUSE	Q64523	0	1E-12
K1C40_MOUSE	Q6IFX3	0	1E-12
UBQL1_MOUSE	Q8R317	0.0008	1E-12
KS6A2_MOUSE	Q9WUT3	0.0008	1E-12
DPOD1_MOUSE	P52431	0.0014	1E-12
HABP4_MOUSE	Q9JKS5	0.0014	1E-12
STAG2_MOUSE	O35638	0.0008	1E-12
MYH7B_MOUSE	A2AQP0	0	1E-12
MYH1_MOUSE	Q5SX40	0	1E-12

Supplementary Table S1. Altered protein expression in GAD67-GFP interneurons 24 h post-TBI. Columns display Uniprot ID, Accession number, False discovery rate (FDR) and the adjusted p-value obtained by a homoscedastic two-sided t-test with Bonferroni correction.

Unipod ID	Accession	FDR	p-Value (adjusted) sham vs contralateral GFP-
MAST1_MOUSE	Q9R1L5	0.0014	1E-12
RHOB_MOUSE	P62746	0	1E-12
K1H1_MOUSE	Q61765	0	1E-12
2ABB_MOUSE	Q6ZWR4	0.0014	1E-12
HPT_MOUSE	Q61646	0.0015	1E-12
CNNM2_MOUSE	Q3TWN3	0	1E-12
TT21A_MOUSE	Q8C0S4	0	1E-12
NGAL_MOUSE	P11672	0	1E-12
K1C12_MOUSE	Q64291	0.0015	1E-12
UBA1Y_MOUSE	P31254	0	0,04165864
RASH_MOUSE	Q61411	0.0014	1E-12
P85B_MOUSE	O08908	0	1E-12
G3PT_MOUSE	Q64467	0	1E-12
TYB10_MOUSE	Q6ZWY8	0	1E-12
K2C6A_MOUSE	P50446	0	1E-12
RB22A_MOUSE	P35285	0.0008	1E-12
KRT35_MOUSE	Q497I4	0	1E-12
RBM4B_MOUSE	Q8VE92	0.0008	1E-12
LAD1_MOUSE	P57016	0.0014	1E-12
NEMF_MOUSE	Q8CCP0	0	1E-12
RL40_MOUSE	P62984	0	1E-12
SI1L2_MOUSE	Q80TE4	0.0014	1E-12

Supplementary Table S2. Altered protein expression in GFP-cells isolated from the contralateral cortex of GAD67-GFP mice 24 h post-TBI. Columns display Uniprot ID, Accession number, False discovery rate (FDR) and the adjusted p-value obtained by a homoscedastic two-sided t-test with Bonferroni correction.

Supplementary Figures

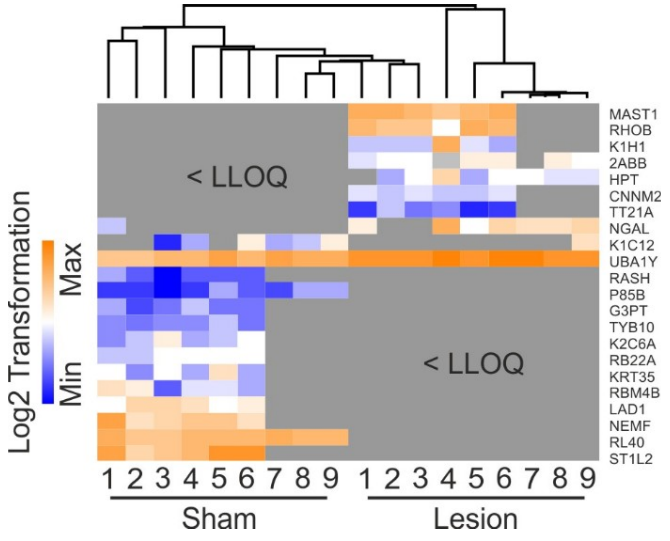

Supplementary Figure S1.: Label free quantification of GFP negative cells in the contralateral hemisphere in GAD67-GFP mice 24 h post-TBI. 22 proteins were identified in GFP- lysates derived from the contralateral cortex of GAD67-GFP animals 24 h post-TBI. None of these regulated proteins was identified in the GFP+ population.

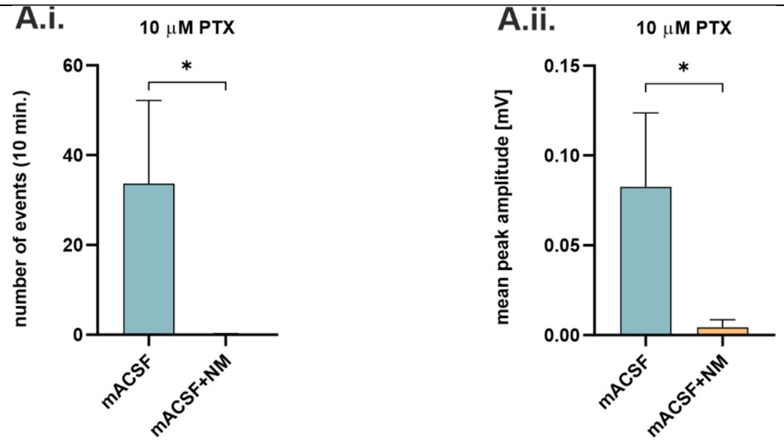

Supplementary Figure S2.: Number of epileptic events and peak amplitudes in presence of 10 µM PTX. Mann-Whitney-U test revealed epileptic events at relatively low concentration of 10µM PTX in the group lacking NM-treatment (NM vs no NM p*=0.0119 for events and peak amplitude), indicating an elevation of the seizure-threshold induced by NM.

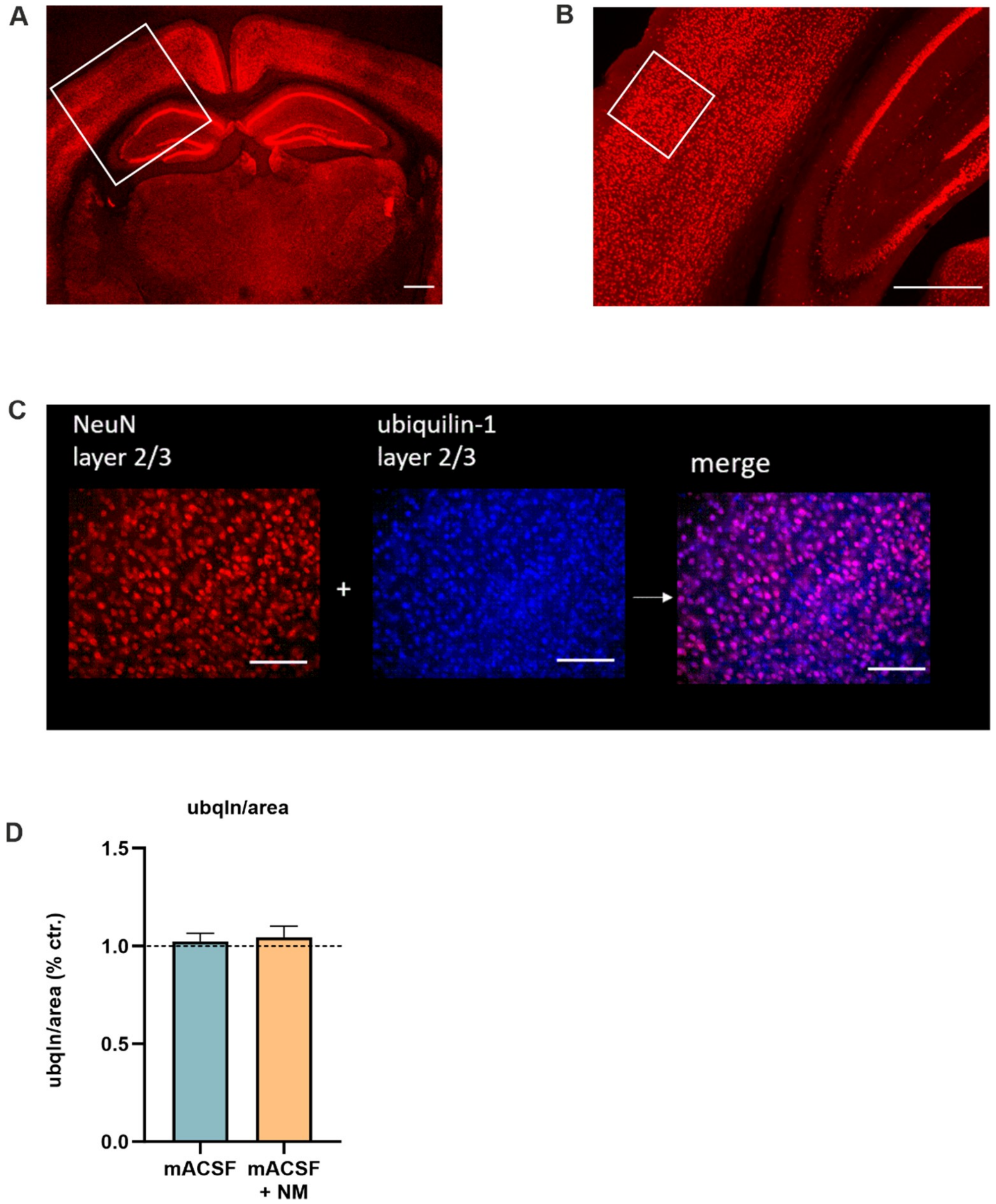

Supplementary Figure S3. Immunohistochemistry of the somatosensory cortex. Immunohistochemical staining of the somatosensory cortex in three C57BL/6n-mice under different experimental conditions (ACSF/ mACSF/ mACSF + NM). The ubqln1 distribution in the somatosensory cortex is displayed in relation to NeuN. **(A)** 1,25x overview of the NeuN staining of 20 µm thick coronal section. The white square indicates the part of the somatosensory cortex, which is magnified in **(B)** (4x). Scale bar = 500 µm. The white square in **(B)** frames the approximate area of interest. **(C)** Comparison and merge of ubqln1 and NeuN distribution in layer 2/3 (20x). Scale bar = 100 µm. Ubqln1 is co-localized with neurons and appears in cells in the background. **(D)** The bar chart revealed no significant difference between the three experimental conditions: control, mACSF and mACSF + NM (n = 3 mice, Kruskal-Wallis test).
